# Serious Gaming Technology in Upper Extremity Rehabilitation: Scoping Review

**DOI:** 10.2196/19071

**Published:** 2020-12-11

**Authors:** Elisavet Koutsiana, Ioannis Ladakis, Dimitris Fotopoulos, Achilleas Chytas, Vassilis Kilintzis, Ioanna Chouvarda

**Affiliations:** 1 Lab of Computing, Medical Informatics, and Biomedical-Imaging Technologies School of Medicine Aristotle University Thessaloniki Greece

**Keywords:** serious gaming, gamification, upper extremity, upper limb, rehabilitation

## Abstract

**Background:**

Serious gaming has increasingly gained attention as a potential new component in clinical practice. Specifically, its use in the rehabilitation of motor dysfunctions has been intensively researched during the past three decades.

**Objective:**

The aim of this scoping review was to evaluate the current role of serious games in upper extremity rehabilitation, and to identify common methods and practice as well as technology patterns. This objective was approached via the exploration of published research efforts over time.

**Methods:**

The literature search, using the PubMed and Scopus databases, included articles published from 1999 to 2019. The eligibility criteria were (i) any form of game-based arm rehabilitation; (ii) published in a peer-reviewed journal or conference; (iii) introduce a game in an electronic format; (iv) published in English; and (v) not a review, meta-analysis, or conference abstract. The search strategy identified 169 relevant articles.

**Results:**

The results indicated an increasing research trend in the domain of serious gaming deployment in upper extremity rehabilitation. Furthermore, differences regarding the number of publications and the game approach were noted between studies that used commercial devices in their rehabilitation systems and those that proposed a custom-made robotic arm, glove, or other devices for the connection and interaction with the game platform. A particularly relevant observation concerns the evaluation of the introduced systems. Although one-third of the studies evaluated their implementations with patients, in most cases, there is the need for a larger number of participants and better testing of the rehabilitation scheme efficiency over time. Most of the studies that included some form of assessment for the introduced rehabilitation game mentioned user experience as one of the factors considered for evaluation of the system. Besides user experience assessment, the most common evaluation method involving patients was the use of standard medical tests. Finally, a few studies attempted to extract game features to introduce quantitative measurements for the evaluation of patient improvement.

**Conclusions:**

This paper presents an overview of a significant research topic and highlights the current state of the field. Despite extensive attempts for the development of gamified rehabilitation systems, there is no definite answer as to whether a serious game is a favorable means for upper extremity functionality improvement; however, this certainly constitutes a supplementary means for motivation. The development of a unified performance quantification framework and more extensive experiments could generate richer evidence and contribute toward this direction.

## Introduction

### Serious Gaming in Upper Limb Motor Rehabilitation

Motor rehabilitation in various parts of the body such as the upper or lower limbs aims to help patients restore dysfunctions that affect their mobility. In this scoping review, we focus on motor disabilities related to the upper extremities. The motivation behind this review was first introduced within one of our group’s research projects related to upper limb rehabilitation, termed “Modern Interface Platform for Motor Control and Learning on People With Motor Disorders” [1-3]. Our purpose was to search the literature regarding upper limb rehabilitation using serious games to provide guidance for proceeding with creation of the project’s platform. With the term “serious games,” we refer to video games created with a purpose other than entertainment, such as education, health care, politics, and engineering. The aim of this study was to review all of the upper limb rehabilitation techniques related to serious games regardless of the cause of motor dysfunctions.

Therapists have developed several clinical methods to indicate motor ability, such as range of motion (ROM) or range of force. In addition, specialized evaluation tests such as Fugl-Meyer Motor Function Assessment (FMA), Action Research Arm Test (ARAT), and Melbourne Assessment of Unilateral Upper Limb Function (MAUULF) aim to estimate the improvement of a patient’s motion condition. The usual rehabilitation scheme consists of repeated motion exercises for a specific body part, with the aim of restoring ability as close to the normal condition as possible.

The idea to introduce gamification to the therapeutic protocol of upper limb rehabilitation was born as a means to motivate patients during the rehabilitation schemes but also represents a new method for monitoring the upper limb motion for further analysis. The first attempts of the introduction of gamification in upper limb rehabilitation appeared in 1999 by a team at Rutgers University [4], making use of a custom prototype robotic arm aiming to map the motion of the palm and wrist with force resistance. This concept was extended with development of a computer-based game that guides the patient to make various movements with the palm and fingers. The same system went through various modifications [5-7], and the latest version of the system was published a few years later [8-11], including significant alterations and improvements regarding the digital environment and the therapeutic approach. Among these early attempts, a study published in 2000 [12] presented a system that uses a robotic device in conjunction with the commercial game Arkanoid for wrist rehabilitation, and another study published in 2002 [13] described an equivalent approach using a resistive joystick.

These rapid technological developments led to more elaborate devices regarding motion capture, challenging researchers in this field to investigate this type of rehabilitation.

### Significance of This Scoping Review

Over the last few decades, there has been an increasing amount of studies regarding the enhancement of rehabilitation with the introduction of new technologies. A systematic review on the implementation of serious games and wearable technology in rehabilitation practices for patients recovering from traumatic bone and soft tissue injuries was published by Meijer et al [14]. Another review attempted to depict the implementations of brain-computer interfaces in the rehabilitation of motor dysfunctions following stroke [15]. Nonetheless, these overviews do not include games specifically developed for rehabilitation or “wearable-controlled” games. Therefore, the primary aim of this scoping review was to summarize the field of upper extremity rehabilitation combined with serious games, providing a map of the research approaches used to date. The main research goals were to: (1) explore the technologies used for upper limb rehabilitation; (2) discover distinct methods, common characteristics, and objectives of these efforts; (3) identify challenges and limitations from these previous efforts; and (4) examine the types of analysis methods used to quantify the treatment outcome.

This effort will contribute to the detection of gaps or limitations in this area, and may lead to new research paths and ideas.

The rest of the paper is organized as follows. The Methods section depicts the procedure that was followed regarding the literature search, data management, and eligibility criteria of this review. The Results section presents the statistical results, including figures, after reviewing the included studies. Finally, the Discussion section comments on the results and delineates possible limitations of this study, along with highlighting the importance of this review for further development of this research area.

## Methods

### Design

In this scoping review, we followed the PRISMA-ScR (Preferred Reporting Items for Systematic Reviews and Meta-Analysis extension for Scoping Reviews) [16] guidelines for the literature search, study selection, and extracted information. We further referred to studies on scoping review methodology, including Arksey and O’Malley [17] and Peters et al [18].

### Literature Search

This review included articles published from 1999 to June 2019. The PubMed and Scopus databases were used for the literature search. The keywords utilized in the literature search were: “rehabilitation,” “hand,” “upper limb,” “upper extremity,” “upper arm,” “game,” “serious gaming,” and “serious game,” which were investigated in titles and abstracts of articles published in the English language. The following search query was used: rehabilitation AND (hand OR upper limb OR upper-limb OR upper extremity OR upper-extremity OR upper arm OR upper-arm) AND (game OR serious gaming OR serious game). Subsequently, duplicated articles were removed, and the remaining studies were screened for eligibility.

### Data Management

Two individual researchers (EK and IL) conducted the literature search and the removal of duplicates, and one author (IL) screened the titles and abstracts for eligibility under advisement by IC. The remaining studies were reviewed by EK, IL, and DF, guided by a set of inclusion and exclusion criteria to extract information from the selected articles. The extracted information followed a structure defined by IC, EK, and IL, as follows: (i) year of publication, (ii) purpose of the study, (iii) part of the upper limb for rehabilitation, (iv) sensors used, (v) disease that led to the patients’ condition, (vi) game type, (vii) game scenario, (viii) game target, (ix) clinic- or home-based application, (x) supervised or unsupervised, (xi) software used for creation of the rehabilitation game, (xii) hardware development, (xiii) system limitation, (xiv) use of a pilot study or not, (xv) number of patients in the pilot study, (xvi) evaluation methodology, and (xvii) features extracted from the game.

**Figure 1:**
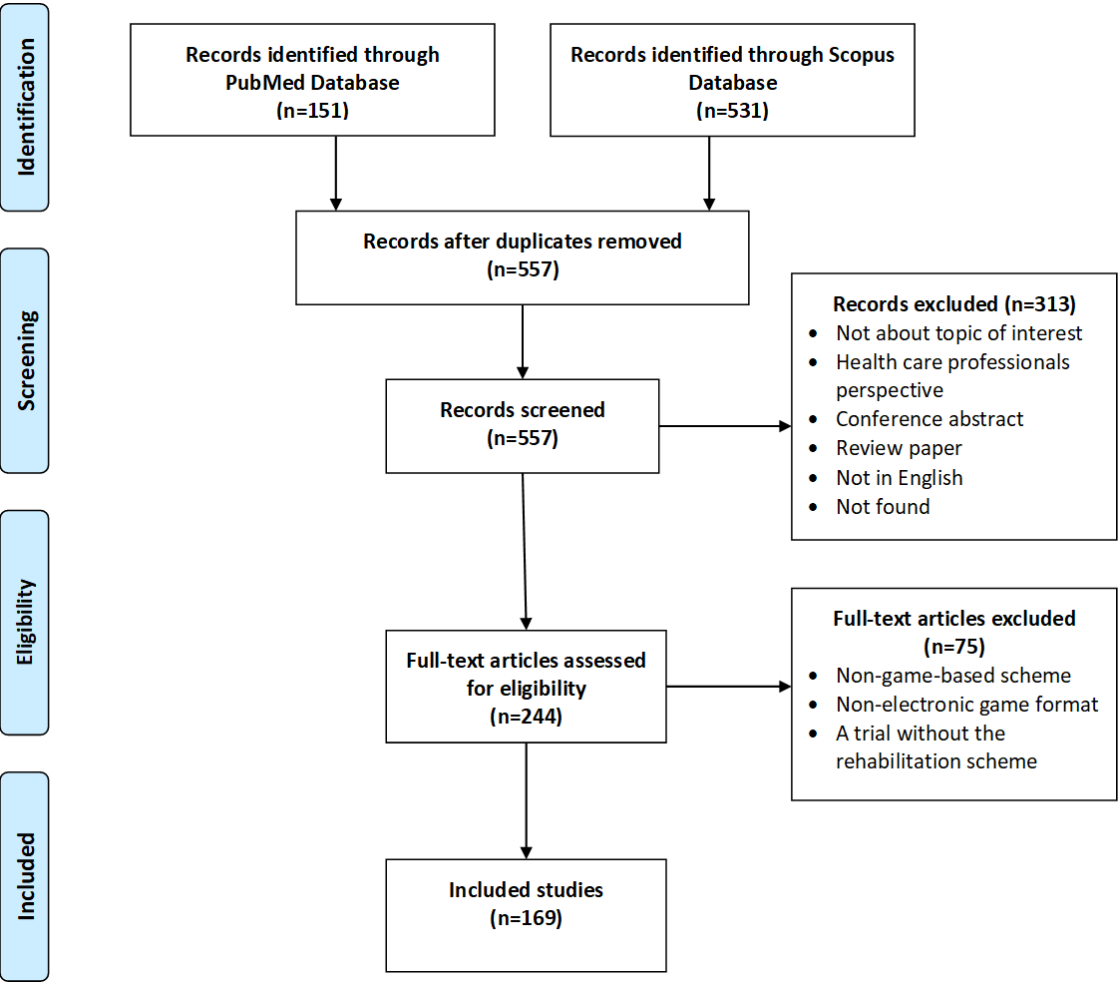
PRISMA-ScR (Preferred Reporting Items for Systematic Reviews and Meta-Analysis extension for Scoping Reviews) [16] flow diagram of the literature search and final included studies.

The literature search was conducted in July 2019 with the requirements described above, and a total of 682 studies were identified, including 151 from the PubMed database and 531 from the Scopus database. After removal of duplicates, 557 studies were screened with the criteria set, resulting in a total of 244 articles. In addition, 75 studies were excluded due to meeting one or more exclusion criteria, and 169 studies were finally included in the scoping review about upper limb rehabilitation based on serious gaming technology. The most common reasons for a study to be excluded were the absence of a serious game from the rehabilitation procedure and the development of a system that did not focus on upper extremity rehabilitation. Figure 1 shows the flow diagram of the exclusion stages for this review.

### Inclusion and Exclusion Criteria

Inclusion criteria for eligibility of the selected articles were: (i) any form of game-based arm rehabilitation (interactive computer-based game, mobile/table app, or platform game software) and (ii) published in a peer-reviewed or conference journal. Exclusion criteria for this review were: (i) a nonserious game–based scheme of rehabilitation with a sensor (only using a sensor or robotic arm, without the accompanying serious game); (ii) a trial of a serious game rehabilitation scheme without any technical description of the game; (iii) medical article based on a health care professional’s perspective for arm rehabilitation without any technical description of a game; (iv) not published in English; or (v) a review, meta-analysis, or conference abstract.

### Synthesis of Results

Based on the extracted information, we created 11 factors of categorization for the data. The extracted information is presented in the Data Management subsection above. The factors were determined based on the combination of the extracted information. All analysis factors were categorical, except for *device development*, which was a Boolean factor. In some cases, studies could belong to more than one category (eg, some studies mentioned analyses on both the wrist and fingers as targeted *upper extremity parts*, while others included both the score and time for the *game target*). Descriptive statistics were used for these factors to present an aggregated view of the studies and percentages.

The results extracted from the included studies are presented according to the following structure: (i) statistics depicted in charts, (ii) descriptive statistics that provide information regarding the tendencies of research efforts, and (iii) conclusions extracted not only from the statistics but also from the general picture formed from the analysis of all included studies.

## Results

### Overview of Extracted Studies and Factors

**Figure 2:**
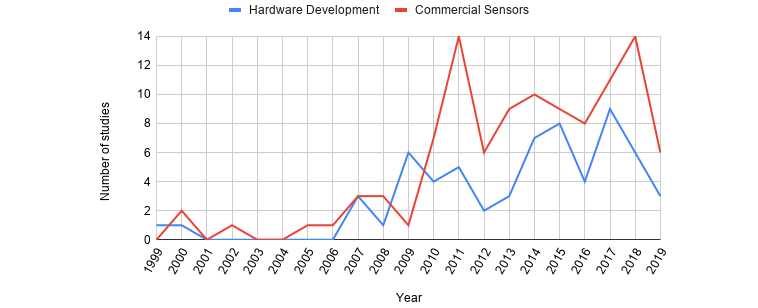
Distribution of the publications over time. The “Commercial Sensors” category refers to studies using commercial sensors or any combination of commercial devices for the rehabilitation scenario, and the “Hardware Development” category refers to studies that created any type of robotic arm, glove, or other device for the connection with the game platform.

Based on our literature search, the first study was published in 1999; however, only a few relevant papers were published in this field up to 2006. In 2007, researchers showed greater interest in upper extremity rehabilitation using new technologies based on serious games, and the number of publications has continued to rise up to the present day. Figure 2 summarizes the studies published on upper limb rehabilitation using serious games over the years. Notably, we only included studies published until June 2019, which means that the line graph in Figure 2 presents only half of the year for 2019. Table 1 summarizes the main factors that were used to draw conclusions and that were further analyzed.

**Table 1:** Factors analyzed in the review.

Extracted information	Description
Medical condition	Underlying categories for the upper limb motion problems: stroke, general motion deficits, cerebral palsy, hemiparesis, neurological motor deficits, Parkinson disease, burn contractures, brain impairment, general cognitive deficits, shoulder injury, and wrist injury.
Upper extremity part	The upper extremity part targeted for rehabilitation: upper extremity/limb, fingers, palm, wrist, elbow, forearm, shoulder, and hand muscles.
Device development	The differentiation between hardware development and commercial sensors. The “commercial sensors” category includes studies that used commercial sensors/devices or any combination of commercial devices for the rehabilitation scenario, whereas the “hardware development” category includes studies that created any type of robotic arm, glove, or other device for the connection with the game platform
Game type	The type of serious game developed: virtual reality, augmented reality, video game, electronic board game, and mobile health apps.
Game target	The game scenario. Task completion (specific scenarios regarding the case, such as follow the line, daily activities, create shapes, collect a number of items), time (complete the level of the game in time intervals), score (increase the level score), force (studied the patient’s force in controlling the sensor).
Sensors	The preferred device for the upper extremity exercise in conjunction with the game.
Hardware use limitations	Limitations regarding the hardware used in the included studies.
Supervision level	The rehabilitation scheme takes place at home or in a clinic while the patient is supervised by an expert or not.
System testing with users: pilots and trials	The testing part of the proposed scenario. Some studies conducted a trial or a pilot trial for testing the rehabilitation system, while others did not. In the latter studies, the trials were conducted with patients, control subjects, or both.
System evaluation	In cases in which the proposed system was tested, there were several means of evaluation: questionnaires, interviews, clinical tests, and scores before and after the rehabilitation scheme.
Extracted game features	The extracted characteristics, using the game, for further analysis: time, game performance, kinematic indicators, range of motion.

### Medical Condition

To specify the *medical condition*, the categories were created based on the references used by the authors of the included papers regarding the medical condition that caused the motor dysfunction. For example, stroke and cerebral palsy are subcategories of hemiparesis or neurological motor deficits. However, some studies mentioned only hemiparesis or neurological motor deficits as a cause of motor dysfunction without any further explanation, while others specified that stroke was the cause of motor disability. Owing to this heterogeneity, we created the categories based on the references for the studied medical conditions. According to our literature search, stroke was the most common reason for upper extremity motor dysfunction and the need for rehabilitation using technology. More than half (56.8%, 96/169) of the studies introduced a system for upper limb rehabilitation after stroke, followed by general motion deficits (29.6%, 50/169 studies). Additional categories with lower frequency in the retrieved literature were cerebral palsy, hemiparesis, and neurological motor deficits. Furthermore, one study was related to Parkinson disease [19], one study was related to burn contractures [20], two studies addressed patients with brain impairment [21,22], three focused on general cognitive deficits [23-25], and other conditions were identified as shoulder [26] or wrist [12,27] injuries.

### Upper Extremity Part

Most of the included studies (48.5%, 83/169) referred to rehabilitation of the upper extremity/limb in general, whereas others (40.2%, 68/169) focused on a specific part such as the fingers, palm, wrist, elbow, forearm, and shoulder.

### Device Development

The results of our search indicated that researchers in this field are showing more interest in commercial sensors that continue to evolve. Less interest is placed on the development of new devices designed for motion of a specific upper extremity part. This may be due to the more costly and time-consuming development of such specific devices. Studies on hardware development accounted for 37.3% (63/169) of the total studies, whereas there was double the number of studies related commercial sensors, representing 62.7% (106/169 studies) of the total. Despite the fewer attempts to address hardware development, there seems to be continuous interest in this research area. However, it is evident that there are more fluctuations of publications over time in the case of hardware development due to the difficulty of the task (ie, the time and knowledge required to create a device), whereas commercial sensor–related studies showed a consistent increasing trend over time (Figure 2).

Between the two *device development* categories, the results regarding representation of the studied *medical condition* and *upper extremity part* factors did not vary in general. Stroke and upper extremity/limb accounted for more than half of all studies related to commercial sensors (56.6%, 60/106 and 48.1%, 51/106 studies, respectively) and hardware development (57%, 36/63 and 51%, 32/63, respectively). Nevertheless, these two categories have several differences concerning the *game type* and *game target* approaches, as described below.

### Game Type

*Game types* were classified into different categories of virtual reality (VR), augmented reality (AR), video game, electronic board game, and mobile health (mHealth) apps. The included studies generally used the term “VR” to refer to games simulating the real world in 3D virtual environments generated by computer graphics (not necessarily using VR headsets), whereas the term “AR” is generally used to refer to two types of games: (1) games showing a real environment, but some objects are enhanced by computer-generated perceptual information; and (2) games representing a virtual world, including the real upper limb of the user (eg, using cameras). Furthermore, “video games” refer to the creation of 2D games, whereas “electronic board games” refer to an interactive table or board. Finally, the “mHealth apps” category includes studies that describe the games as VR or AR, which are health apps using mobile or tablet games.

Based on these definitions, the majority of studies included in the review (74.6%, 126/169) approached the rehabilitation problem by developing VR games. This *game type* offers an alternative reality to the patient, transforming a repetitive exercise of a rehabilitation routine into an amusing and appealing game to spend their time.

The next most common *game type* was video games (15.4%, 26/169), along with some efforts to develop AR systems (10.1%, 17/169). With respect to AR, most of these studies used cameras and markers on the hand to recreate objects on the screen [28-39], although some recent studies used advanced technologies to create a 3D reality [23,40,41].

**Figure 3:**
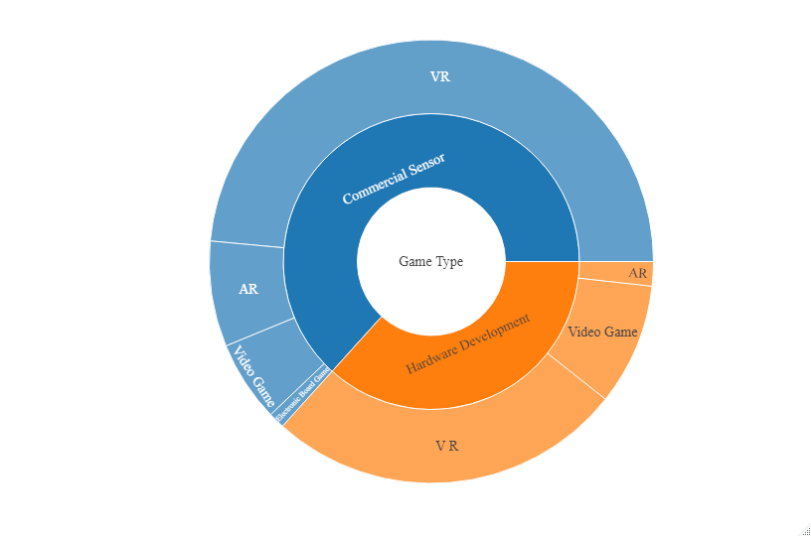
Comparison between the two *device development* categories regarding the *game type*. The ratio of virtual reality (VR), augmented reality (AR), video games, and electronic board games between the *commercial sensors* and *hardware development* categories is shown.

Comparing the two categories of *device development* regarding *game type*, the ratio of VR and video games was proportionally equal (Figure 3). Surprisingly, AR games exhibited essential differences in the two categories, with 7% (14/17) of the AR studies belonging to the category of commercial sensors and only 2%(3/17) belonging to the category of hardware development. In cases of AR, as mentioned above, most researchers used cameras and markers on the hand to capture the movement and incorporate it in the game (ie, combined commercial devices), which explains the higher percentage of studies in the commercial sensors category.

Over the years, technological development has led to increased incorporation of smartphones and tablets in our daily routines. Naturally, researchers have begun testing this new technology in many fields, including upper limb rehabilitation based on serious games by creating mHealth apps. Seven studies [35,40,42-46] created mobile games, and five studies [47-51] developed tablet games in an attempt to study a portable and easy-to-use-anywhere system for patients to perform their exercises. However, the touchscreen is the main component representing the evolution of board games. Two surveys [52,53] studied the use of electronic board games during supervised clinic sessions.

### Game Target

The *game target* classification is summarized in Table 1. The highest percentage in this category was based on the target task completion (62.1%, 105/169 studies) and score (41.1%, 70/169 studies), with both designs focused on training to improve patients’ ability. The approaches for the game scenario did not substantially differ between the two *device development* categories. Most games adopted scenarios similar to the corresponding therapy process and imitated movements from daily activities such as lifting a cup. Thus, the scenario varied depending on the part of the upper extremity that was targeted. Nevertheless, it is evident that in the case of attempts that belong to hardware development, the purpose is focused on more specific (fine) movements (ie, accuracy of the movement achieved in object placement), with noticeable interest in the force that the user exerts [13,47,54-61]. Moreover, another class of *game type* is the time (ie, completion time of the tasks). Overall, 16.6% (28/169) of the studies aimed at achieving time reduction of the specific task, thereby motivating the user to compete with themselves.

### Sensors

#### Commercial Sensors

A variety of commercial or noncommercial devices have been proposed for rehabilitation of the upper extremities combined with serious gaming based on the researchers’ ideas and accessible technologies at the time of publication. The most commonly used *commercial sensor* is the Kinect depth sensor, an accessory developed for the gaming platform Xbox. Kinect seems to be the most preferred sensor for capturing body parts and following their movement in space, which was used by 15.4% (26/169) of the studies included in the review. Some of the studies used only the Kinect sensor for their systems [26,62-73], whereas others combined it with biosignal capturing devices such as electromyogram (EMG) [24,41,74,75] or a sensing jacket [52] to gain better control of the user’s movement for the final goal (ie, rehabilitation). In addition, some studies have used Kinect combined with gaming devices such as VR headsets [76] and a Wii balance board [77] or other devices such as goniometers [78-80], Tyromotion Timo plate [77], Xsen 3D sensor [81], body markers [82], and a customized haptic glove [83]. Furthermore, two studies focused on a different brand of depth sensor for their research, termed PrimeSense [84,85].

With respect to commercial gaming accessories, a few studies focused on individual sensors such as VR headsets [86-88], Wii remotes [89-95], or the P5 glove [96,97] in an attempt to incorporate the existing devices to rehabilitation practices. Another *commercial sensor* that has attracted researchers’ interest is Leap Motion, a hand-tracking sensor, which is most commonly used alone [19,25,40,43,98-103]. One study also combined the Leap Motion sensor and a VR headset [104] in an attempt to create a VR environment for the user as a reinforcement of after-stroke rehabilitation methods. Another study [105] combined the Leap Motion sensor with a thermographic camera and a radiofrequency identification system for body part identification.

Additionally, some studies have attempted to create their own tracking system using *commercial sensors*. In some cases [57,58,78-80,106-110], sets of inertial measurement units (IMUs) were used as basic tracking sensors to measure the body’s force, orientation, and angular rate. The sensors were placed on different parts of the upper limb or body to track the coordinates of the arm and, consequently, the arm movement. Some studies combined IMUs with Kinect to better determine the placement and movement of the body in space.

Overall, 17.8%(30/169) of the studies included webcams and cameras in their systems. Half of them [8,29-31,33,34,37,55,90,94,111-115] used only webcams in an attempt to create a home-based and easy-to-use patient system. The other half used either simple cameras [36] or cameras combined with a marker (ie, glove, card) to track the movement [21,39,116], gaming accessories such as PlayStation controllers [117] and Nintendo Wii remote [89], or a customized exoskeleton glove [118] and an eye tracker [38]. One study also included a motion-capture thermal camera [119] for motion detection in an attempt to avoid holding or wearing any controls or devices, which may be challenging and restrictive for the patient.

Besides interest in developing robotic devices identified in the hardware development category, some research teams have also focused on robotic devices that are already available on the market. Several studies used haptic devices such as Phantom Omni [22,120-122], Novint Falcon [77,122,123], Haptic Master [59,124-127], and Geomagic Touch [128], whereas others used robotic gloves such as CyberGrasp [124,126,129] and 5DT Data Glove [130,131] or robotic arms such as Barrett Wam [132] and Armeo Spring [20,108,133].

**Figure 4:**
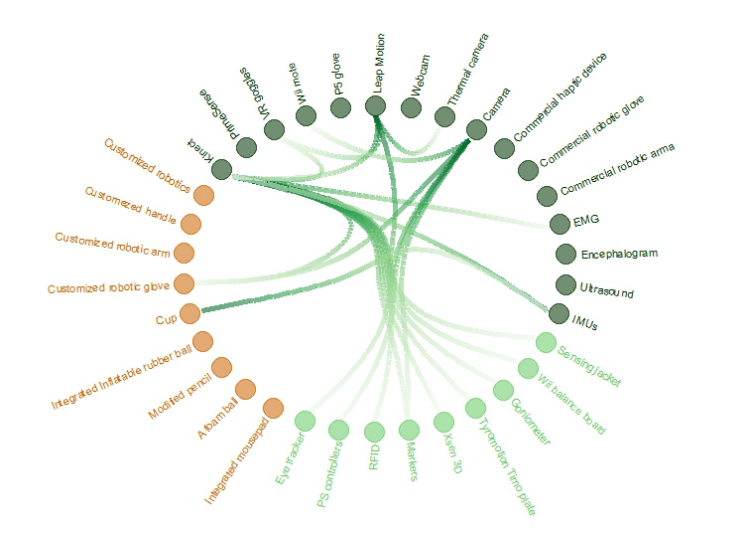
Overview of the devices used in the included studies. The green nodes indicate the sensors included in the *commercial sensor* category, while the orange nodes are those included in the *hardware development* category. The dark green nodes represent sensors that have been used individually or in combination with one of the sensors represented by the light green nodes.

In addition, some studies attempted to either control or monitor patients’ movements using medical devices such as EMG [30-32,34,41,44,87,112,134-142]. With EMG, it is possible to monitor how the muscles respond to nerve signals. In this way, physicians could observe the patient’s upper extremity motion to prevent risky movements or to be sure that the patient is controlling the arm in the right direction based on his/her rehabilitation scheme. Moreover, some researchers have investigated the use of standard medical devices in serious gaming rehabilitation systems, including encephalogram [142-144] to monitor brain activity and ultrasound to estimate finger force [145]. A summary of the devices and their different combinations used to date is presented in Figure 4.

#### Hardware Development

Several different ideas of *hardware development* have been put forward in the developed devices. There are simple approaches that included objects such as a cup [54], combinations of objects and sensors such as a cup with IMU sensors [57], custom-made devices representing daily movements for elderly patients [146], a mousepad integrated with a CD motor [147], a foam ball and a modified pencil [148], and an inflatable rubber ball with an air pressure–sensing device [149]. Additionally, there are more complicated and time-consuming approaches identified in this category, including developments of exoskeleton robotic devices that focus on specific parts of the hand. These are studies in which robotic gloves were developed to control the fingers [35,42,61,79,150-156], and in which robotic arms were created to cover the surface from the shoulder to the wrist [4-11,48,49,110,139,157-161]. Additionally, many research teams have focused on the development of a handle to obtain control of the arm force and movement [12,56,135,162-173] or similar robotics [174-177]. A summary of these devices is shown in Figure 4.

### Hardware Use Limitations

Despite the use of advanced technology, many studies that used commercial sensor devices mentioned limitations regarding the used hardware. For example, various studies [41,62,65,69,80,82] mentioned the possibility of Kinect’s dysfunctionality in detecting movements or parts of the body. Moreover, several studies mentioned poor body part detection using different commercial sensors, such as poor hand detection from the Leap Motion device [103], poor detection with use of a camera [28], and poor detection in the combined use of the Leap Motion sensor with Oculus Rift VR goggles [104].

Limitations were also mentioned with respect to the *hardware development* category, including the need for enhanced calibration or upgraded components for better monitoring of accurate data. One study reported the need to improve a control strategy [139], while others mentioned general hardware issues [61,109,134,156,166]. In addition, some studies referred to the need for adjustments regarding the range of motion of the users [49,160] or the size of the hand [5]. Finally, one study pointed out difficulties in the use of the hardware system due to the poor design (ie, it was difficult for the user to put on the robotic glove and thus use it) [140].

### Supervision Level

In general, the *supervision level* involved with each system was not always evident. Approximately half of the articles did not mention the home or clinical use of their systems, while some mentioned various supervision levels. It is worth noting that only 42.6% (72/169) of the included studies mentioned any supervision level for their proposed system; 3%(5/18) of the unsupervised cases belonged to the *hardware development* category and 8%(13/18) belonged to the *commercial sensors* category.

### System Testing With Users: Pilots and Trials

All of the included studies described a system that has been developed by the corresponding research team. Some of them included small pilot or limited-range trials, while others were complemented by subsequent studies reporting the results of pilot surveys. It is notable that the most frequent limitation in cases with no pilot study was the absence of clinical trials and the deficient testing of the system. Overall, 33.7%(57/169) of the studies tested their system with healthy subjects and therapists, whereas 27.1%(46/169) did not test the system at all. Although the remaining one-third of the research teams conducted clinical trials with patients, the majority of them included a very small number of patients, and also included healthy subjects in some cases to enlarge the sample. Among the 169 papers included in this scoping review, there were 2291 participants in the pilot and clinical trials. However, from the total number of participants, 689 were patients and 1602 were healthy volunteers, clinicians, therapists, and researchers.

### System Evaluation

The *system evaluation* methods could not be easily categorized. This is because the research teams chose vastly different approaches for evaluation of their proposed system based on the target of the study and the available means at the time of publication. In 11.8% (20/169) of the included studies, no means of evaluation were mentioned regarding the introduced system, whereas 23.7% (40/169) of the studies seemed to focus only on users’ or therapists’ feedback about user experience via questionnaires and interviews. In these attempts, therapists and clinicians were given the opportunity to try the system with respect to the rehabilitation goals, the game’s environment, and devices’ safe use before being tested by patients.

Furthermore, 29.6%(50/169) of the studies used metrics regarding functional recovery via standard tests such as FMA, ARAT, and MAUULF; scores such as ROM for the elbow and forearm; and the Jamar strength test for strength of the hands. By using standard tests and scores as evaluation methods, researchers can measure the progress of a patient regarding motion dysfunction before and after the rehabilitation scheme. In addition, several studies tried to extract game features to introduce quantitative measurements for evaluation of patient improvement. Among these studies, 65%(49/75) did not analyze the evaluation methods, referring to them more generally as “data analysis” and providing descriptive statistics or as “monitoring data” in which the sessions were recorded using several sensors. In the next section, we discuss an extended analysis regarding the *extracted game features* of the studies.

### Extracted Game Features

Quantitative measurements of the treatment outcome are critical for clinical rehabilitation practice, which constitute an objective method for evaluating the patient’s medical progress. With these measurements, physicians can closely monitor the therapy process and adjust the treatment protocol individually. Among the studies included in this review, only a few described an assessment process of the patient’s recovery status based on extracted features.

Some of these studies [21,65,98,124,126,128,137,147,155,164] used the time category *game target*, which was used to define metrics. These metrics were mainly classified into categories of hand movements or the duration, task, session completion of gestures, and reaction time.

Game performance was another consistent feature among the studies. In some cases, performance was associated with the score, and in other cases it was associated with task completion of the game target. Several studies [21,73,98,101,126,155] collated the score of the extracted features with standard clinical metrics (eg, box-and-block test and FMA) and suggested a strong correlation between them. By contrast, one study [98] reported that game achievements (the score regarding the number of coins collected) are not always an objective indicator of a patient’s therapy progress. In addition, the task completion extracted features for a group of studies is the result of a patient’s performance compared with a gold-standard method, which is usually an ideal movement trajectory (perhaps executed from a healthy subject) that the patient should follow, or an arithmetic measurement calculated after quantification of a specific hand movement [128,132,164,174]. Deviations from the gold standard are calculated and constitute the extracted features. For example, Lioulemes et al [132] first classified hand trajectories from patients with a support vector machine classifier and a hidden Markov model, and then calculated their deviations from the optimal trajectory as errors in space and time. For the second part of their analysis, they used dynamic time warping, which is a method for aligning optimally time-dependent sequences.

It is worth noting that several studies monitored other kinematic indicators of the patient’s health condition that are not included in the *game target* classes for describing the patient’s overall improvement [21,68,124,126,128,135,137,164]. Specifically, five studies [68,124,128,137,164] referred to the smoothness of the hand movement, or hand steadiness (jerk), during therapy sessions as the main feature. This jerk behavior is mainly described as abrupt changes in the direction of the hand’s motion, and the way it is calculated may slightly differ from one study to another regarding the mathematical procedure employed. Furthermore, two studies [43,68] mentioned the use of hand trajectory curvature as a feature for kinematic analysis. In one study, the curvature of the hand trajectory was calculated as its deviation from an ideal straight line [178], whereas the second study computed the logarithm of the median of path curvature [179] to quantify “motion irregularity.” Both studies included trajectory curvature in their criteria for measuring the arm’s coordination. In addition, three studies [5,124,126] focused on fractionation as a game feature. Specifically, fractionation describes the ability to isolate the movement of the fingers and volitionally activate the motor units of the hand. Finally, two studies [136,137] focused on the muscle activation and caption of functional movement.

ROM and data regarding the angles of the hand during its motion constitute another significant group of features that have been commonly used by researchers and health professionals to quantify therapy progress. Several studies [12,20,69,81,98,103,152] referred to the calculation of these kinds of features. Four studies [20,69,98,152] reported that they monitored ROM data (minimum, maximum, and average) for each single joint or exercise movement, and only one study [12] calculated ROM as a summative score of multiple movements or the difference. This extracted feature comprises a valuable tool, as it can be compared across sessions, subjects, or between the impaired and nonimpaired limb of each subject. According to one research team [98], plots of features regarding ROM facilitate the detection of distraction or movement pain during a patient’s therapy session, thus resulting in a more effective performance diagnosis. Table 2 presents a summary of the *extracted game features.*

**Table 2:** Summary of the extracted game features.

Categories, Features		Number of studies
**Time**		
	Time-related	10
**Game performance**		
	Score/task completion	6
	Golden standard comparison	4
**Kinematic indicators**		
	Hand jerk	5
	Trajectory curvature	2
	Fractionation	2
	Muscle activation	1
**Range of motion**		
	Range of motion	7

It should be noted that this scoping review does not report every metric for each study, as our purpose was not to elaborate on how each study implemented the assessment of the patient’s improvement but rather to outline and categorize the features extracted from the motion analysis process, excluding metrics of patients’ engagement and motivation. Furthermore, in this attempt of feature extraction categorization, no distinction was made between studies that evaluated these features with a group of patients and those that conducted trials with healthy subjects.

## Discussion

### Principal Findings

With this scoping review, we aimed to explore the trends associated with deploying technologies for functional rehabilitation of the upper extremities. The results indicate that there has been increasing interest in these applications over time. The rapid evolution of technology contributes to new approaches concerning clinical practice and personalization of therapies. There is currently a wide range of sensors available for capturing motion (eg, Kinect, Leap Motion, IMUs) along with attempts to translate these technologies into an environment projected on computer screens, in VR headsets, or in AR image processing. The capabilities of various sensors related to motion, in conjunction with serious gaming, are used by research teams to develop contemporary systems for both doctors and patients.

One of the main advantages of this study is the overview of the current state of the field of upper extremity rehabilitation using serious games. As part of our research interest, we tried to investigate this topic to better understand the various approaches used to date. Based on this summary, we present a set of characteristics that depict a common direction and provide a complete picture of the sensors and technologies utilized to achieve the therapy purpose in terms of standard clinical practice.

The results indicate increasing research interest in the domain of serious gaming deployment in upper extremity rehabilitation. Based on the descriptive analysis, we can examine different aspects of this field of research. In particular, stroke seems to be a common *medical condition* for many research teams to trigger a study about upper limb mobility. This is understandable considering that stroke is the third leading cause of disability worldwide [180].

With respect to the factor *upper extremity part*, we found only one study published in 2016 by Hung et al [134] that presented a home-based rehabilitation system focused on the hand muscle. In addition, 26 of 36 studies that included the fingers as the hand part of focus used *commercial sensors* for their proposed rehabilitation system.

While reviewing the surveys included in our literature search, it is a safe assumption that computer graphic development in the last few decades has led to generalization of the term “VR.” In 1992, Coates [181] defined VR as follows: “electronic simulations of environments experienced via head-mounted eye goggles and wired clothing, enabling the end-user to interact in realistic three-dimensional situations.” Most of the studies included in this scoping review referred to their systems as “VR systems,” but did not consider the original definition quoted above. These studies instead addressed a more generalized notion of VR that includes all systems that can simulate the real world (via 3D virtual environments generated by computer graphics) and use sensors—but not necessarily VR headsets—for their interaction framework with the users. Besides VR systems, we found three studies that referred to their *game type* as “mixed reality” [42,62,89], which is a tabletop AR platform mixing VR, AR, and electronic board games for the proposed game.

With respect to the *game target*, every choice of game scenario was related to different aspects of rehabilitation schema. The game scenario combines the targeted upper extremity part, the moves that the patient needs to repeat for training, and the researcher’s goal for an outcome that entertains and motivates the user. Increases in task completion and score are the most common scenarios differing in the content of the task based on the rehabilitation scheme.

Additionally, there is a broad selection of *sensors* and their combinations, as presented in Figure 4, that can be used for the creation of various rehabilitation systems. The most commonly combined sensors are Kinect, Leap Motion, and a camera, which seemed to be the most preferred devices over time, as described in further detail below. Technological development has provided researchers with more and more tools for testing their ideas, leading to new efficient implementations. Besides these new sensors, during the past decade, smartphones and tablets have entered our daily lives, and have rapidly become an integral part of life. Since 2011, researchers have been testing their capabilities in conjunction with upper extremity rehabilitation. It is worth noting that the wrist and fingers were the most commonly targeted *upper extremity parts* for rehabilitation using mobile or tablet app–based games, mainly because of the touchscreens.

In this study, we classified the used devices according to the *commercial sensors* and *hardware development* categories. Comparing the two *device development* categories, both showed differences in the proposed implementations. The larger number of publications related to the *commercial* *sensors* compared to the *hardware development* category implies greater interest in growing an idea of a game based on an advertised device. This higher interest may occur because it is more time-consuming or expensive to develop a new device than to explore the applications of already existing brands. Nevertheless, in the *commercial sensors* category, some studies reported poor hand part detection during sensor use, as elaborated upon in the Hardware Use Limitations subsection, deploying problems in practice. In addition, in the *hardware development* category, researchers have used the opportunity to develop a device based on the targeted hand part; however, depending on the case, a customized device could raise problems such as difficulties in use and adjustments for every hand size. Furthermore, many of the rehabilitation schemes included in the *commercial sensors* category targeted a home-based system using portable devices. However, for the *hardware development* category, the fragile and limited customized devices require supervised use, which poses a challenge for home-based trials.

**Figure 5:**
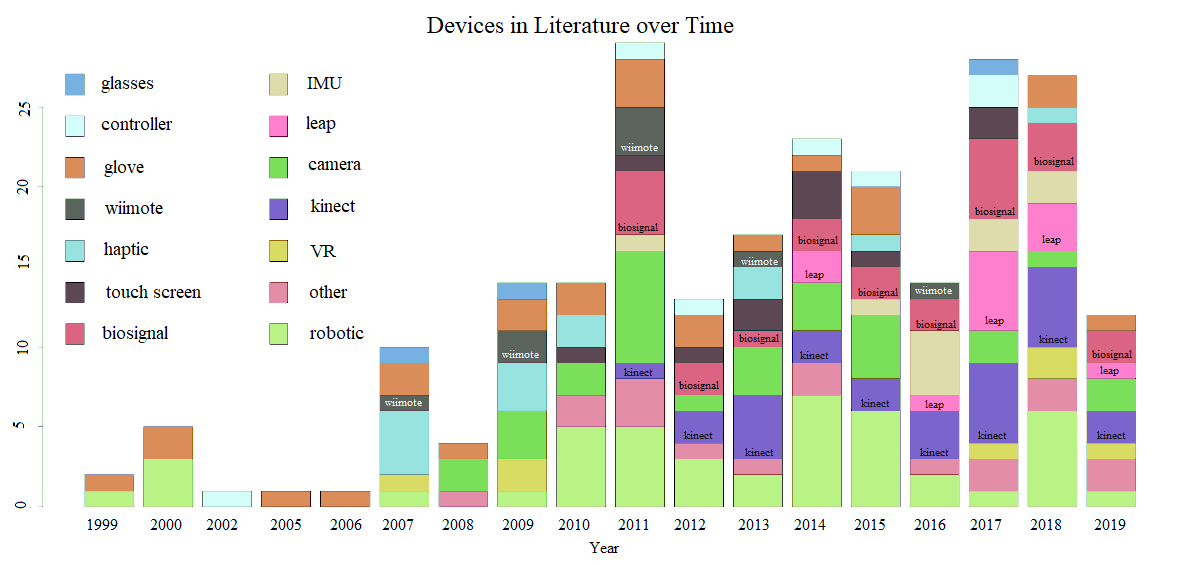
Summary of devices reported in the literature over time. Groups of sensors used for studies of upper limb rehabilitation are presented in a timeline. The groups include devices from both the *hardware development* and *commercial sensors* categories.

Figure 5 presents a timeline of the devices reported in the literature over the past three decades. All of the categories presented in this figure include devices that may belong either to the *hardware development* or *commercial sensors* category. The aim of this figure is to present the use of every individual sensor in the research on upper limb rehabilitation over time. Since 1999, researchers have extensively studied glove sensors and robotics, a category including arms and handles. In addition, it is evident that since the first release of Kinect in 2010, there has been continuous interest in its use in upper extremity rehabilitation over the years. It is worth noting that until the release of Kinect, several studies were using the Wii remote, with the majority published in 2011, whereas after this point, there were only a few such attempts reported in 2013 and 2016. By contrast, Kinect gained increasing interest from 2011 to 2019. The Leap Motion sensor was first released in 2010, but the first attempts to use it in upper limb rehabilitation were only reported 4 years later in 2014, and the highest number of papers published in this field appeared 3 years later in 2017. Moreover, an inverse relation was observed between studies published on the Kinect sensor and the use of cameras over the years. Finally, the category biosignals includes biosensors such as EMG and electroencephalogram. Since 2011, these sensors have been consistently used in many studies (Figure 5). 

Based on our results, one-third of the introduced systems conducted clinical trials with patients to test their implementation. Although one-third of the included studies constitutes a sufficient number of attempts, in most of these cases, there is a need for a higher number of subjects and better testing of the rehabilitation scheme efficiency over time.

Concerning the *evaluation* of the proposed systems, many studies did not include any reference about the evaluation process of the patient’s health or have not provided sufficient evidence about the assessment of the system. This is probably because most of this research was conducted at an early stage of development, prior to any related clinical trials, or because the researchers only aimed to introduce an idea about a rehabilitation system, mentioning their observations related to technical aspects as a secondary aim. Nevertheless, system evaluation of the tested rehabilitation schemes has typically been conducted using questionnaires or interviews, individually or in combination, about the game and the experience in general, medical standard tests examining improvement of the motion, and extracted game features about motion analysis or game performance. Notably, 68.7%(101/147) of the studies that included some form of assessment for the introduced rehabilitation game mentioned, among others, user experience as a factor for the system’s estimation. In addition to user experience assessment, the most common system evaluation method involving patients was the use of standard clinical tests. Efforts for the creation of quantitative measurements of game-based treatment constitutes an attempt to provide evidence about the efficiency of the rehabilitation scheme and to personalize clinical practice.

*Game features* such as visual feedback of user actions and reward mechanisms via score/points or goal achievement were present in all rehabilitation gamification attempts. These features, which are an essential part of one of the biggest industries of the present day (ie, the game industry), are known to induce user engagement and are a core part of the rationale behind the gamification of health treatment protocols. In this regard, an increase in patients’ interest as a motive for investing in gamified approaches was taken for granted in most cases, although some studies also provided results from questionnaires that confirmed the above assumption.

### Limitations

The broadness of the field of upper limb rehabilitation using serious games constitutes a limitation leading to many potentially included studies for this review. There was a significant number of studies, each suggesting different ways to approach the rehabilitation scheme but with poor sources or minimum attempts. Many conference papers have been published over the years introducing thoughts and preliminary results, but with no further analysis and implementation of their idea for rehabilitation. Although our exclusion criteria limited the range of the existing literature to some extent, this review includes several uncompleted attempts. Moreover, since we used specific keywords such as “upper-extremity,” “rehabilitation,” and “serious game” in different combinations, in an attempt to focus on the area of interest, we concede that some surveys in the field may have been excluded. Nevertheless, we are confident that the remaining studies that met all of the inclusion criteria can reflect the state of the field of upper extremity rehabilitation employing serious games, thereby assuring the reliability of our conclusions.

In addition, a limitation of this study is the lack of categorization based on gross motor vs fine motor or testing usability vs testing effectiveness. The many differently structured papers in combination with the heterogeneity in the provided information made such categorization very complicated and led us to the decision not to include these categories. It may be worth analyzing these categories separately to obtain an overview of this field in a different study.

Finally, due to the rapid technological progress, we consider another limitation to be the fact that this review includes studies only published up to June 2019.

### Conclusion

Upper extremity motor dysfunction is a common problem that requires rehabilitation. Researchers studying the engagement of patients to the rehabilitation schemes have established several ways to develop more amusing training sets to better motivate patients. Technological progress constitutes an ally of these attempts, allowing for the combination of a traditional rehabilitation routine with serious games. In the last two decades, there has been a significant number of publications regarding upper limb rehabilitation using serious games, which is a field that continues to evolve based on user experience. Our goal regarding this review was to provide a complete overview of the field based on published studies over the years. Overall, this scoping review highlights several facts that point to the usefulness of serious games in rehabilitation in future medical procedures, as well as several weaknesses and challenges that have to be addressed. Despite the numerous attempts for establishing and evaluating game-based rehabilitation systems, more evidence is needed considering such systems not only as a means for patient motivation but also as an actual means for achieving upper extremity functionality improvement. In this vein, despite the challenges in the generalization and comparability of specific game decisions and implementations, it is important to support the efforts for the creation of quantitative measurements of game-based treatment, performance and outcome, and build evidence of its clinical value. In this direction, it would be important to work toward creating a framework for the therapeutic use of such gamified approaches, including the optimal dosage, personalization means, adaptations over time, session performance assessment, and therapeutic outcome. Such a therapeutic framework could enable the synthesis of more solid clinical evidence around game-based treatment, and eventually its incorporation in the clinical routine.

## Abbreviations

AR: augmented reality
ARAT: Action Research Arm Test
EMG: electromyogram
FMA: Fugl-Meyer Motor Function Assessment
IMU: inertial measurement unit
MAUULF: Melbourne Assessment of Unilateral Upper Limb Function
mHealth: mobile health
ROM: range of motion
VR: virtual reality
